# Alkali Lignin-Based Biopolymer Formulations for Electro-Assisted Drug Delivery of Natural Antioxidants in Breast Cancer Cells—A Preliminary Study

**DOI:** 10.3390/ijms26157481

**Published:** 2025-08-02

**Authors:** Severina Semkova, Radina Deneva, Georgi Antov, Donika Ivanova, Biliana Nikolova

**Affiliations:** 1 Department of Electroinduced and Adhesive Properties, Institute of Biophysics and Biomedical Engineering, Bulgarian Academy of Sciences, 1113 Sofia, Bulgariabiliananikolova2000@yahoo.com (B.N.); 2 Laboratory of Genome Dynamics and Stability, Institute of Plant Physiology and Genetics, Bulgarian Academy of Sciences, 1113 Sofia, Bulgaria; antov8107@abv.bg; 3 Department of Pharmacology, Animal Physiology Biochemistry and Chemistry, Faculty of Veterinary Medicine, Trakia University, 6000 Stara Zagora, Bulgaria; 4 Department of Chemistry and Biochemistry, Faculty of Medicine, Trakia University, 6000 Stara Zagora, Bulgaria

**Keywords:** breast cancer, alkali lignin, biopolymer formulations, electroporation, drug delivery, anticancer activity, natural antioxidants

## Abstract

Recently, a number of natural biologically active substances have been proven to be attractive alternatives to conventional anticancer medicine or as adjuvants in contemporary combination therapies. Although lignin-based materials were previously accepted as waste materials with limited usefulness, recent studies increasingly report the possibility of their use for novel applications in various industrial branches, including biomedicine. In this regard, the safety, efficiency, advantages and limitations of lignin compounds for in vitro/in vivo applications remain poorly studied and described. This study was carried out to investigate the possibility of using newly synthesized, alkali lignin-based micro-/nano-biopolymer formulations (*Lignin@Formulations/L@F*) as carriers for substances with antioxidant and/or anticancer effectiveness. Moreover, we tried to assess the opportunity for using an electro-assisted approach for achieving improved intracellular internalization. An investigation was conducted on an in vitro panel of breast cell lines, namely two breast cancer lines with different metastatic potentials and one non-tumorigenic line as a control. The characterization of all tested formulations was performed via DLS (dynamic light scattering) analysis. We developed an improved separation procedure via size/charge unification for all types of *Lignin@Formulations*. Moreover, in vitro applications were investigated. The results demonstrate that compared to healthy breast cells, both tested cancer lines exhibited slight sensitivity after treatment with different formulations (empty or loaded with antioxidant substances). This effect was also enhanced after applying electric pulses. *L@F* loaded with Quercetin was also explored only on the highly metastatic cancer cell line as a model for the breast cancer type most aggressive and non-responsive to traditional treatments. All obtained data suggest that the tested formulations have potential as carriers for the electro-assisted delivery of natural antioxidants such as Quercetin.

## 1. Introduction

Well-recognized approaches for cancer treatment in modern medicine include various surgical techniques, as well as the administration of radiotherapy, chemotherapy, targeted therapy, hormone therapy, and immunotherapy. Appropriate therapy methods are chosen depending on the tumor type, location, and stage, as well as on the health status of the patients [1].

Despite advances in oncology, each of the mentioned approaches faces ongoing challenges, and their efficacies remain limited. For example, similar to conventional anticancer drugs, newly synthesized agents with amplified cytotoxicity against cancer cells, including elaborated protein kinase inhibitors, have shown drug resistance, insufficient tumor accumulation, and the induction of toxic side effects on healthy tissues due to suboptimal bio-distribution [2]. Recently, the efforts of scientific researchers have been focused on developing new strategies for cancer treatment, in which natural compounds, possessing anticancer activity, combined with chemotherapeutic drugs could affect cancer cell proliferation and help to reduce tumor growth [3,4,5,6,7].

In parallel to new cancer treatment approaches, nanomedicine is also a novel and progressive therapy direction, focused on alternative drug delivery and improving treatment efficacy while reducing detrimental side effects to normal tissues [8]. The leading engineering principles while elaborating nanomedicines include the following: (i) designed to be biocompatible and biodegradable; (ii) developed to enhance the pharmaceutical properties of the therapeutic compounds to improve their therapeutic efficiency and overcome drug resistance; (iii) delivering cargoes in a target manner to a certain cell or tissue, thus decreasing their side effects; and (iv) the possibility to control or trigger drug release upon a specific stimulus [9]. Therefore, it seems that the primary material(s), used for designing nanoparticles, must be strictly selected regarding their bio-organic properties.

With the progress in biomedicine, the applications of “green” biomaterials and renewable raw materials in medicine have been paid increasingly close attention due to their low cost, natural origin, and eco-friendly properties. Lignin is the most abundant natural polyphenol that builds plant cell walls and the second most abundant biopolymer in nature (15–30%) after cellulose [10,11,12]. It is generated as a waste product from paper mills and biorefineries that process lignocellulosic biomass into fuels and chemicals [13]. Generally, natural biopolymer possesses a complex and undefined structure, closely related to the type of extraction method used and the subsequent modification [14,15]. Structurally, lignin contains three basic phenylpropanoid monomers, in which different functional groups, such as many phenolic and aliphatic hydroxyl groups, carboxylic groups, and methoxy groups, can be identified and impact its antioxidant properties [16,17]. In the past ten years, scientists have focused on studying the antioxidant properties of lignin variations, which, nowadays, also continue to be investigated regarding cell cultures [18,19,20,21]. Due to its relative nontoxicity, biodegradability, redox activity, stability, and low nominal price, lignin is an ideal macromolecule for use in developing micro-/nanostructures with promising potential for biomedical applications [22,23,24,25]. Additionally, the many active groups, located on the lignin surface, enhance the possibility of its bio-functionalization processing through forming interactions with various water insoluble chemotherapeutic drugs, as well as synthetic antioxidants and bioactive compounds with anticancer activities [26]. Our previous studies also show that extra modification of lignin is considered to be a method for carrying bioactive compounds to specific organs [27,28]. The application of redox active compounds, derived from various plant sources, as bio-functionalizing molecules is increasing in popularity in anticancer research due to the possibility of inducing target therapy and the ability to initiate a natural immune response and minimize the toxic side effects on healthy cells and tissues [29,30,31,32,33,34]. Numerous molecular mechanisms such as the modulation of cell cycle signaling (MAPK, PI3K, NF-κB pathways), the activation of intracellular antioxidant enzyme activities, the influencing of energy and redox-homeostasis, etc., have been identified as key characteristics for inducing apoptosis and/or decreasing cancer cell proliferation activity [35]. Despite significant progress in the study of bioflavonoids and their role in cancer therapy, some physicochemical properties, such as low water solubility, light and heat instability, etc., still compromise their bioavailability [36,37,38].

In this study, alkali lignin was used to prepare micro-/nano-carriers for the bioflavonoids Quercetin, morin and naringenin, and their sizes were subsequently improved. Optimizing the size of the micro-/nano-formulations is crucial for ensuring experimental consistency and avoiding negative effects on cell vitality. Achieving optimal size during their formulation is still a difficult task for developing drug delivery systems. Moreover, concentration determination for the in vitro application after purification procedures is an even greater challenge in the case of biopolymer formulations, composed from units without spectral characteristics. On the other hand, the insufficient number of scientific studies on the therapeutic effects of lignin carriers of bioactive substances (vitamins, bioflavonoids) applied to cancer and non-cancer cell lines influenced the goals and objectives of the present investigation. The proposed in vitro preliminary study on breast cancer and non-cancer cell lines aimed to verify the safety, efficiency, and potential anticancer activity of the novel “green” synthesized lignin-based formulations. In addition, the electroporation technique, combined with alkali lignin formulations for enchanting their anticancer activity, was applied.

## 2. Results and Discussion

Although scientific studies have recently been published that demonstrated the antioxidant, antiviral, antibacterial, and other activities on different types of lignin and lignin-based formulations [39,40,41], the insufficient information available about the specific biological effect(s) of lignin on cells, such as its affinity with the cell wall, impact on cellular energetic metabolism, activation/inhibition of intracellular signaling pathways, etc., continue to be of significant interest for scientists. Furthermore, studies on their antitumor activity are still under active investigation, prompting our preliminary study.

### 2.1. Synthesis of Alkali Lignin-Based Submicron Formulations

Due to its complex chemical structure, lignin can be used for preparing stable lignin particles [42,43]. The various techniques used for lignin particle preparations have been elaborated, including antisolvent precipitation, solvent exchange, aerosol processes, supercritical solvent precipitation, ultrasonication, interfacial polymerization/cross-linking, etc. [44]. In our study, the complexes of pure alkali lignin and alkali lignin encapsulated with bioflavonoids were produced using the self-assembly properties of biopolymer in a water/ethanol mixture. The self-assembly properties of lignin exist because of the presence of various functional groups and their capability to initiate spontaneous interactions such as hydrogen bonds, electrostatic interactions, van der Waals forces, and π-π interactions between aromatic rings. The formation of the listed chemical bonds assists for determining the composition of lignin’s molecular aggregates, with suitable properties, geometric forms, and dimensions, essential for arranging micro-/nanoparticles with biomedical application [45,46]. The corresponding synthesis methods of the unloaded and loaded alkali lignin particles, as well as their detailed characterization, including their initial size, total phenolic content, relative concentrations of acidic and basic active sites/functional groups on the surface, and in vitro cumulative release of flavonoids in simulated gastrointestinal enzyme-free media, were previously investigated and reported by us [27]. The experimental data have proved the existence of a higher concentration (2.037 particles/μL) and average size (approximate 6.1 μm) and an over 2.3 times increased total phenolic content in bioflavonoid-loaded particles compared to unloaded ones. In vitro cumulative release has shown that the quantity of the released flavonoid in simulated small intestinal medium was twice that released in the simulated colon environment and had three times the release efficiency of that determined in the stomach. Therefore, the lignin-based formulations have shown potential for efficient use to load poorly water-soluble compounds and to improve their release profiles at pH 5.5 and 7.4 in a sustained manner [47,48]. Additionally, the stability of the synthesized alkali lignin submicron formulations was tested by us based on their role as a function of time, salt concentration, and pH. In pure water at room temperature, alkali lignin particles were stable for more than a month, but low-pH environments or high salt concentration induced aggregation. Similar properties for the self-assembly of lignin particles have been reported for the lignin nanoparticles obtained from kraft lignin pulping, enzymatic hydrolysis lignin, dioxane lignin, and alkali lignin [46,49,50,51].

### 2.2. DLS Data and Electroporation Influence on Formulation’s Structure

After synthesis procedures, all types *L@Formulations* were additionally characterized via DLS analysis before and after the separation procedure performed via Vivaspin^®^6-centrifugation, as described above in “Materials and Methods”. The estimated data for size and ζ-potential of *L@F* are presented in Table 1.

It is clearly shown that the ζ-potential values for all types formulations are negative, which is the best possible scenario for a hypothetical in vitro application, considering the natural negatively charged state of cell membranes. All estimated initial sizes are between 300 and 500 nm, and it is correct to accept all formulations as synthesized in a micro-ranged scale. After executing the proposed unification procedure via centrifugation, for all types of formulations, we estimated a more than two times greater size decrease and approximately the same difference in the ζ-potential value, as is shown in Table 1. The lower measured dimensions and preservation of the negative charges of alkali lignin-based formulations after separation encourage us to consider them as carriers with a promising potential. Moreover, the DLS-analyzed parameters predict an opportunity for good rates of electro-assisted internalization via electroporation.

Based on this, the next part of the investigation involved verifying whether electric pulses with different intensities influence the formulations’ sizes and ζ-potential values. The expected changes for *L@F* (empty or loaded with Quercetin) before and after applying electrical pulses are shown as an example of the influence of electroporation in Table 2.

In general, using electric pulses to internalize substances/carriers or nanoparticles into cells is partially designed to avoid negative influence on their structure, charge and effectiveness [52,53,54]. The experimental procedure of the electro-assisted cell membrane passage process should be optimized before performing further in vitro tests. Thus, the first step was to select correct treatment parameters to guarantee the preservation of the structural integrity and loading capacity of the formulations. In our case, we found that the size of empty formulations decreases with increasing voltages of applied electrical pulses (Table 2). A near two-fold decrease was observed in the case of treatment with the highest applied voltage (1000 V/cm). The formulation’s charge remained negative despite the applied high-intensity pulses. Similar results (with the same tendency) were obtained for samples loaded with Quercetin alkali lignin-based formulations. It is clear that the lower-intensity (between 100 and 500 V/cm) applied electrical pulses were more suitable for conducting subsequent in vitro experiments because they were found to have a more “unaggressive” formulation structure.

**Figure 1:**
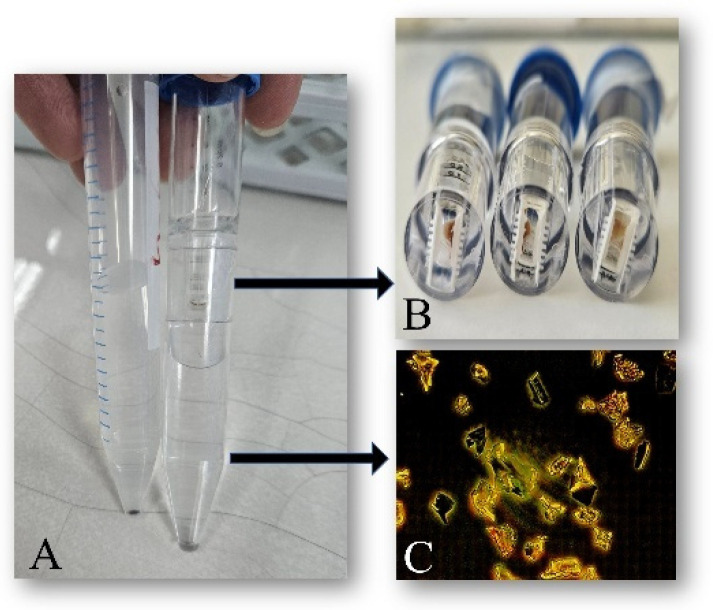
Visualization of proposed separation procedure improvement: (**A**) comparison between normal 15 mL centrifugal tube (left tube) and Vivaspin^®^ 6 concentrator (right tube) after low-speed centrifugation; (**B**) retained high-molecular-weight *L@F* and subunits/comparts in 0.2 µm filters of used Vivaspin^®^ 6 concentrators after centrifugation; (**C**) microscopy images of lower-phase (filtrate) content (magnification 100×).

The estimated data, collected after the proposed separation and unification procedure, are shown in Table 3. All relative concentrations of different *L@F* samples determined via a simple mathematical formula were lower than the initial concentrations, in full agreement with the main reasoning behind the procedure, namely that micro-ranged particles and bigger free subunits and/or comparts are retained in the filter during centrifugation (Figure 1). The explained calculation should be accepted as a multistep procedure before conducting hypothetical in vitro applications, considering the heterogeneity of the initial solutions.

**Figure 2:**
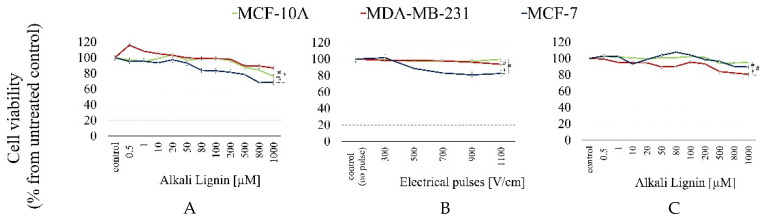
Cell viability 48 h after treatment with the following: (**A**) pure alkali lignin substance (concentration range from 0.5 to 1000 µM); (**B**) electrical pulses with different intensities (300–1110 V/cm); (**C**) combined treatment—300 V/cm and alkali lignin at different concentrations. Green lines—the control “normal” MCF-10 A cell line; red lines—the highly metastatic MDA-MB-231 cell line; blue lines—the low-metastatic MCF-7 cell line. All data are presented as means ± SD from three independent experiments (* *p* < 0.05 MCF-7 cell versus MDA-MB-231 cells; # *p* < 0.05 versus MCF-10A normal cells shows the most statistically significant differences).

The first step of our in vitro preliminary study involved clarifying the effect of breast cell viability after treatment with pure subunits of the proposed formulation—alkali lignin. The results for the cytotoxicity of the tested concentrations of alkali lignin (in a range from 0.5 to 1000 µM), explored via an MTT assay, are presented in Figure 2A. Moreover, we evaluated the cytotoxic effect on the same three cell lines after electro-treatment with electrical pulses of different intensities (Figure 2B), as well as after combining electro-treatment and pure alkali lignin (Figure 2C). The data were examined 48 h after treatment, and the survival rate of cells was calculated as a percentage of the untreated control.

The viability of untreated cells, accepted for 100% of cases, was used as a negative control in cytotoxicity analysis. As shown in the data presented in Figure 2, the survival of all three types of breast cell lines after treatment with an alkali lignin was not reduced by 50%. The heist cytotoxic effect (31.49% cell viability decrease) was observed for MCF-7 cells after applying 1000 μm of alkali lignin (Figure 2A). Statistically significant differences in survival rates between untreated controls and treated samples were reported only for concentrations in the range of 80–1000 μm. For the cancer MDA-MB-231 cell line, a slight increase in proliferation was observed after treatment with lower substance concentrations (0.5–20 μm), but with an increasing alkali lignin concentration, cell survival gradually decreased, with values remaining higher than those measured for MCF-7 cells. A possible explanation for the noticed stimulatory effect is the possibility of the alkali lignin reducing reactive oxygen species (ROS) to levels that promote mutagenesis and cell proliferation [20,55], as well as differences in the metastatic potential of breast cancer lines.

In conclusion, for both tested cancer cell lines, the less aggressive and less metastatic MCF-7 line was more affected by alkali lignin treatment. Moreover, we observed a minimal decrease in MCF-7 cell survival with statistical significance between the non-electroporated controls and the electro-treated samples in the applied electric field intensity range of 500–1100 V/cm (Figure 2B). Total survival was reduced with the increased intensity of the applied electric field, but it did not exceed 18% (MCF-7 cells and 1100 V/cm), probably due to the safety of the used electrical pulses, as well as the reversibility of the pore formation process.

Based on the results, we proceeded with a combined treatment protocol, in which applying electrical pulses with an intensity of 300 V/cm was chosen to ensure that there was no impact on cell viability, combined with selected concentrations of alkali lignin. The data demonstrated slightly increased proliferation for MCF-7 and MCF-10A cell lines compared to MDA-MB-231, which could again be at least partially explained by the antioxidant capacity of alkali lignin molecules.

**Figure 3:**
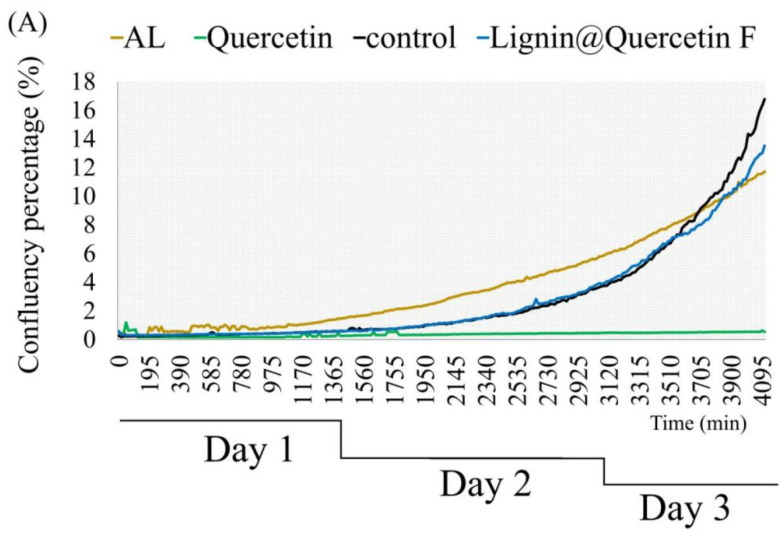

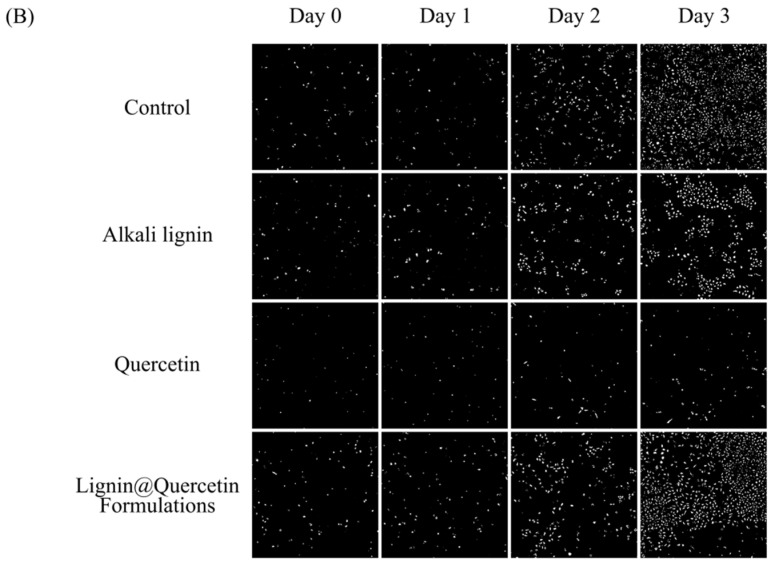
(**A**) The time evolution of cell proliferation on a 2D substrate for three days; (**B**) brightfield lens free microscopy images of 2D cell cultures treated with different substances and followed for three days. The cells in (**B**) were highlighted using the local thresholding method proposed by Phansalkar et al. [59] with a window size of 10^6^ × 10^6^.

In fact, the observed lack of effect on the triple-negative cell line encouraged our study to go further by investigating the anticancer efficacy mechanism of action and hypothetical possibilities for the delivery of natural antioxidant substances by alkali lignin-based formulations, using the triple-negative MDA-MB-231 cell line. Thus, in the next part of this study, we performed brightfield lens-free microscopy via a holographic device to quantify cell proliferation rates, using it as a second methodology to ensure the repeatability of our results. The recorded images and proliferative curves 3 days after treatment are presented in Figure 3, and 2D video records of cell movements and proliferation are shown in the Supplementary Material. The choice of natural antioxidant (Quercetin) as an example of loaded substances was not random. Based on the intensively investigated and well-known anticancer effectiveness of Quercetin on breast cancer cells [56,57,58], we decided to use it for additional research after it was loaded into the empty *L@F* (*L@Quercetin Formulations*). The treatment scheme included control untreated cells, alkali lignin-treated cells (250 µM), Quercetin-treated cells (300 µM), and cells treated with separated by size and charge *L@Q F* samples separated by size and charge (calculated relative concentration: 3.5 µg/mL). We found differences in the proliferation profiles (Figure 3A). The highest proliferative rate was recorded for the untreated control cell, as expected. On the other hand, applying pure alkali lignin stimulates cells to proliferate in a clear time-dependent manner, but at a lower rate compared to control cells. In accordance with the literature data, treatment with Quercetin shows the strongest effectiveness—cells proliferate the slowest. Interestingly, after treatment with *L@Q F*, we found increased cell proliferation with approximately the same tendency as the control until the second recording day. After that, proliferation slowed down, and at the end of the third day, the final cell number count was lower than that of the control but high compared to alkali lignin-treated cells. After 48 h incubation, proliferation changes became notable, with even partial leakage of Quercetin affecting the integrity of the *L@Q F* structures at this time-point and activating their antiproliferative effectiveness. Proliferation rates, some morphological differences, and cell movement fluctuations after treatment are shown in the recorded movies and in the Supplementary Material (Supplementary Movies S1–S4).

**Figure 4:**
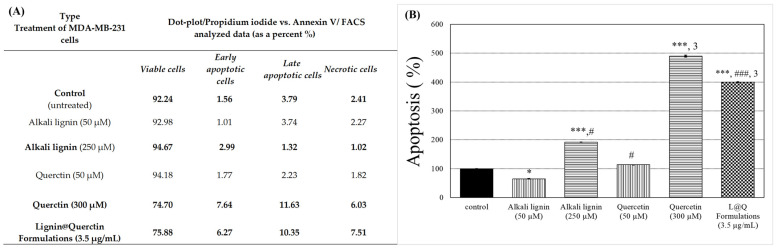
(**A**) Flow cytometric dot-plot for analyzed data of Annexin V-FITC/PI, represented as a percentage; (**B**) flow cytometric Annexin V-FITC/PI analysis of apoptosis in the triple-negative, tumorigenic, highly metastatic MDA-MB-231 induced by alkali lignin, Quercetin, or *Lignin@Quercetin Formulations* for 48 h of treatment. Apoptosis bar graphs represent mean ± SD, n = 2. Levels of statistical significance: * *p* < 0.05; *** *p* < 0.001 control versus alkali lignin (50 µM), alkali lignin (250 µM), Quercetin (300 µM), and *L@Q Formulations* (3.5 µg/mL); # *p* < 0.05, ### *p* < 0.001 between alkali lignin (50 µM) and alkali lignin (250 µM), as well as *L@Q formulations* (3.5 µg/mL); ^3^ *p* < 0.001 between alkali lignin (250 µM) and Quercetin (300 µM), as well as *L@Q Formulations* (3.5 µg/mL).

To understand, even partially, the mechanism of action of the tested substances, the next part of this study investigated a possible connection with cell death processes. Here, flow cytometry analysis of samples from MDA-MB-231 cells analogically treated with substances, Annexin V-FITC, and propidium iodide staining for apoptosis/necrosis was performed. All calculated data are shown in Figure 4, and images from the analyzer are shown in Supplementary Figure S1. Treatment with high-dosage alkali lignin (250 µM) increased the percentage of cells undergoing early apoptosis and led to a percentage decrease in late apoptotic cells compared to the untreated control cells. The highest increase in early apoptotic cells was reported for treatment with higher doses of Quercetin, which is in good correlation with data obtained from cytotoxicity tests [60,61,62]. In the literature, there are a number of analogical observations. For example, decreased cell proliferation and an increased rate of apoptosis through caspase activation after Quercetin treatment of human papillary thyroid cancer cells was reported by Mutlu Altundağ, Ergül et al. [63]. Treatment with *L@Q F* shows a similar trend for apoptosis induction, but it is still lower that pure Quercetin. This observation additionally assumes that the leakage of the lignin-based carrier after treatment and anticancer effectiveness are related to the induction of apoptosis.

**Figure 5:**
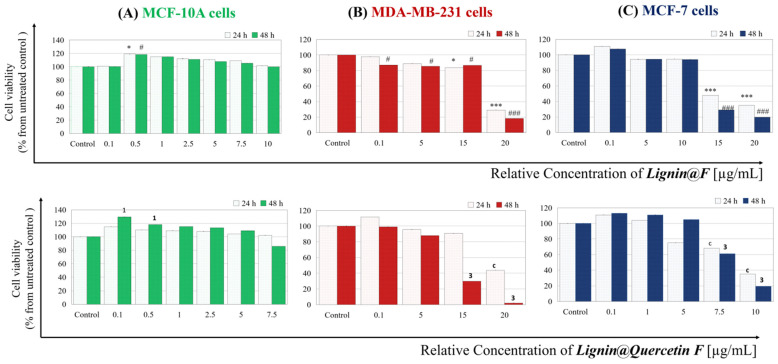
Cell viability after treatment of (**A**) non-tumorigenic control “normal” MCF-10 A cell line; (**B**) highly metastatic MDA-MB-231 cells and (**C**) low-metastatic MCF-7 cell line. Empty *Lignin@Formulations* (upper three graphics) and *Lignin@Quercetin Formulations* (lower three graphics). The light colored columns represent the data for 24 h of treatment and the dark ones represent 48 h. Levels of statistical significance: * *p* < 0.05; *** *p* < 0.001 control 24 h versus *Lignin@F* at concentrations of 0.5 µg/mL, 15 µg/mL, and 20 µg/mL; # *p* < 0.05; ### *p* < 0.001 control 48 h versus *Lignin@F* at concentrations of 0.5 µg/mL, 0.1 µg/mL, 5 µg/mL, 15 µg/mL, and 20 µg/mL; ^c^ *p* < 0.001 control 24 h versus *Lignin@Quercetin F* applied at concentrations of 7.5 µg/mL, 10 µg/mL, and 20 µg/mL; ^1^ *p* < 0.05; ^3^ *p* < 0.001 control 48 h versus *Lignin@Quercetin F* applied at concentrations of 0.1 µg/mL, 0.5 µg/mL, 15 µg/mL, and 20 µg/mL.

In the final part of this study, we try to answer a question: Do the proposed empty and Quercetin-loaded alkali lignin-based formulations influence the cell viability of used breast epithelial cell lines? Therefore, we performed an MTT assay after treatment with carriers separated by size and charge. The results from the conventional assay are presented in Figure 5.

The data show that for the non-tumorigenic control “normal” MCF-10 A cell line, both types low-dosage *L@F* treatments stimulate cell vitality after 48 h. For MDA-MB-231 and MCF-7 cell lines, it was found that concentrations below approximately 10 µg/mL of *L@F* or *L@Q F* cause a cell viability decrease but only within the 20% limit. Nevertheless, low-metastatic cells seem to be more sensitive to those types of substances. The observed decrease in cell viability after applying higher tested concentrations can be explained—the solvent used for initially diluting composed carriers was deionized water. In fact, the concentration range (0.1 to 20 µg/mL) was chosen based on two different points: (i) the limitation of the calculated relative concentration after the separation procedure for each type of carrier is different due to the carriers’ heterogeneity; (ii) since the stock solution of the formulations is aqueous, to treat the cells with it, it is necessary to replace a certain volume of culture medium with the aqueous solution of the tested formulations. Thus, we replaced 1–25% of cell medium volume with Milli-Q^®^ in an additional experiment (data are shown in Supplementary Figure S2) and observed that the upper limit for substituting the cell medium with water solvent is 22.5%, because higher dilution (more than 25%) results in significantly decreased cell viability. This indicates that, in general, an application of *L@F* of the concentration in the range of 5–10 µg/mL (where dilution does not exceed 22.5%) is an appropriate choice for in vitro drug delivery experiments.

**Figure 6:**
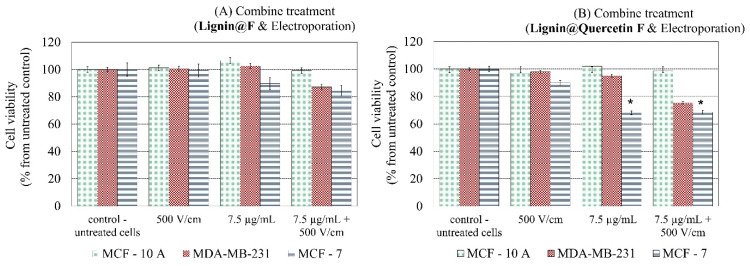
Cell viability after combined treatment of breast cell lines with (**A**) empty *Lignin@Formulations* and electroporation (500 V/cm) or (**B**) *Lignin@Quercetin Formulations* and electroporation (500 V/cm). The green columns show data for the non-tumorigenic control “normal” MCF-10 A cell line; the red columns show data for the highly metastatic MDA-MB-231 cells; the blue columns show data for the low-metastatic MCF-7 cell line. All data are means ± SD from three independent experiments (with * *p* < 0.05 showing the most statistically significant differences).

Finally, we investigate the cytotoxicity effectiveness after combined treatment with electroporation and both *L@F*-empty and Quercetin-loaded samples (Figure 6). The highest statistically significant cell viability reduction (31.55%) is observed after Quercetin application on MCF-7 cells, and the effect is enhanced after combination with an additional 500 V/cm of electroporation. The control non-tumorigenic MCF-10 A cell line remains unaffected, and for the triple-negative MDA-MB-231, a slight decrease (up to 25%) in survival is noted, with clear additivity when applying electric pulses (a 19.7% decrease after applying 500 V/cm was estimated for MDA-MB-231 cells).

All data demonstrate and predict good opportunities for the electro-assisted drug delivery of natural antioxidants such as Quercetin for newly synthesized alkali lignin-based formulations.

## 3. Materials and Methods

### 3.1. Synthesis of Alkali Lignin-Based Submicron Formulations (Lignin@Formulations)

The method used for synthesizing the pure (empty) and natural flavonoid-encapsulated alkali lignin-based submicron formulations used for the below analysis was performed according to our recently reported protocol [25]. The synthesis techniques were based on the alkaline lignin self-assembly method, with water used as an alternative and non-toxic solvent and ethanol and citric acid as cheap and environmentally friendly cross-linking agents. Thus, the method could be classified as a “green” synthesis method. All solid chemicals, such as alkali lignin (CAS number: 8068-05-1) and the flavonoids morin (CAT number: 654055-01-3), naringenin (CAT number: 67604-48-2), and Quercetin (CAT number: 117-39-5), used for synthesizing lignin-based submicron particles, as well as ethyl alcohol, pure ≥ 99.5% (GC) (CAT number 64-17-5), were purchased from Sigma-Aldrich, Darmstadt, Germany. The lactic acid (CAT number 131808) needed for synthesis was purchased from Chimtex Ltd., Dimitrovgrad, Bulgaria. In brief, 125 mg of alkali lignin was dissolved in 25 mL of ultrapure water to a obtain final concentration of 5 mg/mL. Then, 1 mL of pure ethanol (EtOH) was slowly added while stirring the lignin solution with a magnetic stirrer at 500 rpm for 3 min at room temperature. The estimated final concentration of ethanol in the synthesis of alkali lignin-based formulations did not exceed 1%. The mixture was dropwise-supplemented with 7 mL of 1% lactic acid (cross-linking agent), using a syringe, at a flow rate of approximately 4 mL/min, and stirring continued for a further 10 min. At the end of mixing process, the brown clear solution was transformed into a cloudy light brown suspension of submicron particles. Synthesis was conducted via centrifugation (15,000× *g* for 30 min at temperature 10 °C) and triple-washing with ultrapure water. Finally, the microparticles were ultrasonically homogenized twice (4 min each at an intensity of 96%) and lyophilized in a freeze-dryer (at a temperature of −64 °C).

To prepare flavonoid-encapsulated alkali lignin-based submicron formulations, the bioflavonoids morin (0.08 g in 1 mL of EtOH), naringenin (0.04 g in 1 mL EtOH), and Quercetin (0.04 g in 1 mL of EtOH) were used individually. The polyphenol stock solutions were gently added into 5 mg/mL of alkali lignin and stirred using a magnetic stirrer at 500 rpm for up to 20 min. After appending the cross-linking agent, the mixtures were centrifuged, washed, homogenized, and lyophilized, following the parameters described above.

### 3.2. Improved Separation Procedure

An improved size-based separation procedure for unifying *Lignin@Formulations* was proposed. It is characterized by using low-speed centrifugation with Vivaspin^®^6 centrifugal concentrators (Sartorius (Bohemia, NY, USA), Sigma Aldrich (St. Louis, MO, USA)/MERCK (Rahway, NJ, USA)) equipped with PES membrane (0.2 µm pore size). In general, these concentrators (spin-filters) are used for adjusting macromolecule concentration and buffer exchange procedures in research. In our study, aliquots of prepared solutions of each type tested *L@Formulations* diluted with Milli-Q^®^ water (0.05 mg/mL concertation), which were centrifuged via Vivaspin^®^ 6 tubes at a speed of 3000× *g* (4 °C) for 40 min using a swing-bucket rotor with carriers holding 15 mL tubes. The upper phase (supernatant) contained high-molecular-weight *L@F* and particles larger than 0.2 µm, but in the lower phase (filtrate), formulations were smaller than the pore size. Both type solutions were collected for subsequent dynamic light scattering (DLS) analysis, but only filtrate solutions were tested for in vitro applications.

### 3.3. Dynamic Light Scattering (DLS) Analysis

Dynamic light scattering and electrophoretic light scattering techniques were used to determine the size and charge (ξ-potential) of all tested particle compositions using a Zetasizer Nano ZS analyzer (Malvern Instruments, Malvern, UK). Data were averaged from three independent experiments performed in a U-shaped cell with gold-plated electrodes at 25 °C. DLS analysis was performed on the samples for all types of solution—Milli-Q^®^ water solvent, initial stock solutions of all tested *L@F* (0.05 mg/mL) samples, Vivaspin^®^6-separated filtrate solutions, and electroporated solutions.

### 3.4. Calculation of Relative Concentration

We proposed a basic calculation procedure for estimating the relative concentrations (C^R^) of all *L@Formulations* before their application in the in vitro experiments. In brief, all tested micro/nano-formulation solutions were separated via Vivaspin^®^6 centrifugal concentrators and, using a test probe from the initial and filtrates phases, were DLS-analyzed to determine size and ξ-potential, as described above. The ξ-potential of the solvent (Milli-Q^®^ water) was also measured. All calculations were performed based on the estimated DLS data for the ξ-potential of solutions via the following equation:

C^R^ = (U − Z)/(Y − Z) × X

where C^R^ is the relative concentration of the filtrate phase solution for the in vitro application; Z is the DLS-measured ξ-potential of the solvent, used for initially diluting the formulations; Y is the DLS-measured ξ-potential of the initial supernatant phase solution before the Vivaspin^®^6-based separation procedure; U is the DLS-measured ξ-potential of the filtrate phase solution after the Vivaspin^®^6-based separation procedure; and X is the initial solution concentration in mg/mL before Vivaspin^®^6-based separation (0.05 mg/mL). All estimated relative formulation concentrations were fixed as stock solution concentrations of *L@F* filtrate solutions and used in subsequent in vitro assays and tests, which are described below.

It is important to note that the proposed alkali lignin-based formulations are highly heterogeneous structures, so the proposed protocol, which included (i) the separation procedure, (ii) DLS-analysis, and (iii) calculating the relative concentration, should be considered a multistep before each in vitro application experiment, as explained below.

### 3.5. Cell Lines and Culture Conditions

All experiments were performed on a panel of three human breast epithelial cell lines purchased from the American Type Culture Collection (ATCC) (Manassas, VA, USA). As a control line was used, the non-tumorigenic breast epithelial cell line (MCF-10A) was cultivated in Dulbecco’s Modified Eagle’s Medium (DMEM) (Sigma Aldrich/MERCK, USA) supplemented with 10% fetal bovine serum (FBS), 1% sodium pyruvate, 1% MEM Non-Essential Amino Acids (NEAAs), 20 ng/mL of human epithelial growth factor (hEGF), 10 μg/mL of insulin, and 0.5 μg/mL of hydrocortisone without antibiotics. The adenocarcinoma cell lines (MCF-7 and MDA-MB-231) were cultivated in DMEM supplemented with 10% FBS, 1% sodium pyruvate, and 1% NEAA without antibiotics. All cell lines were grown in an incubator at 37 °C and a humidified atmosphere with 5% CO_2_.

### 3.6. Cytotoxicity Assessment

The cytotoxic activities of the different substances investigated on the cultivated cells were measured via the conventionally used MTT [3-(4,5-dimethylthiazol-2-yl)-2,5-diphenyl tetrazolium bromide] colorimetric test [64]. The method is based on the intracellular metabolism of tetrazolium salt for insoluble formazan crystals. The cells were grown in 96-well plates at a density of 1 × 10^5^ cells/mL. After overnight incubation, the cells were treated with different concentrations of the tested compounds (alkali lignin, *L@F*, or Quercetin) with/without electroporation (EP) and incubated for an additional 24/48 h. The tested concentrations were between 0.5 and 1000 µM and 10 and 300 µM for alkali lignin and Quercetin, respectively. All used “relative” concentrations for biopolymer formulations were in a range of 0.1–30 µg/mL, estimated after calculation based on the DLS analysis of ζ-potential, as described above (“Improved separation procedure” and “Calculation of relative concentration”). Later, the MTT solution (10 µL of 5 mg/mL, Sigma-Aldrich, USA) was added to each well, and the plates were re-incubated for an additional 3–4 h. Finally, to solubilize the formed formazan crystals, 100 µL of lysis buffer (10% SDS, 0.01M HCI) was added to each well. The amount of formazan absorption was registered using a microplate reader (TECAN Infinite^®^ 200 PRO, Männedorf, Switzerland) at 570 nm. The untreated cells were used as control probes. The influence on cell viability for each tested compound was calculated using Microsoft Excel and/or Origin (Version 9.6.5) software. All cytotoxicity assessment experiments were performed in triplicate, i.e., at least three times independently.

### 3.7. Electroporation (EP) Protocol

All electro-treatment experiments were performed via a Chemopulse IV electroporator. Flat parallel stainless-steel electrodes with a 1 cm intra-electrode distance were used for applying bipolar pulses. Chemopulse IV equipment includes a large degree of voltage control in the range of 100–2200 V, simplified operations, locking against illegal manipulations, and enhanced protection against electrical hazards. All biphasic pulses used for electro-treatment had the following parameters: a 50 + 50 μs duration, a 20 μs pause between both phases, and an 880 μs pause between bipolar pulses. Different types of cells (1 × 10^5^ cells per well) were seeded 24 h before electroporation to allow complete adherence. All tested compounds in defined concentrations were added immediately before pulse application. In this study, electric pulses with intensities of 300–1100 V/cm were applied. After incubation, the samples were additionally analyzed via cytotoxicity assay, as described above. The controls were treated under the same conditions, but without electric pulse application and/or the addition of substances.

Previously, we executed other types of experiments connected with investigating electroporation effects on the size and ζ-potential of *L@F*, using electric pulses with the same intensity (300–1100 V/cm). The tested formulation solutions were electro-treated in 12-well plates and later analyzed via DLS, as described above.

### 3.8. Cell Proliferation Analysis via Lens-Free Microscopy

High-metastatic MDA-MB-231 cells were plated into 6-well plates at an initial concentration of 2 × 10^4^ cells/mL and incubated at 37 °C and in a humidified atmosphere with 5% CO_2_ for adhesion for at least 2/3 h. After incubation, the cells were treated with different concentrations of the tested compounds (alkali lignin, *L@F*, or Quercetin) at defined concentrations. The lens-free holographic device *Cytonote 6W* (Iprasense, Helioparc, Pau, France) was used to quantify cell proliferation and to track and record 2D cell migration. Image acquisition began 1 h after cell treatment. Images were recorded every 15 min for 72 h. The device has a large recording field of view (29.4 mm^2^). After incubation, all data were analyzed via HORUS (version 6.22.2) and Excel software and presented as proliferation rates and images at different time-points. Additionally, movies presented the 2D cell movements and proliferation rates of differently treated cells, as shown in the Supplementary Material.

### 3.9. Flow Cytometry Analysis (FACS Assay)

In brief, the MDA-MB-231 cells were seeded in 6-well plates at a density 1 × 10^5^ cells/well for full adhesion. After overnight incubation, they were treated with previously calculated and fixed concentrations of the pure subunit biopolymer (alkali lignin), antioxidant substance (Quercetin), or prepared combine formulation (*Lignin@Quercetin Formulations*—*L@Q F*) and incubated for another 24 h in full culture media. Non-adherent cells were washed with PBS at pH 7.4. Following incubation, the cells were detached through trypsinization and labeled with Annexin V-FITC and PI dyes from the ApoFlowEx^®^ FITC detection kit (Exbio, Prague, Czech Republic) according to the manufacturer’s instructions. Finally, the samples were immediately analyzed using a CyFlow^®^ Space SYSMEX tool (Sysmex Europe, Norderstedt, Germany). The FACS assay was executed twice, and the analysis was performed with MACS Quantify Software (version 3.0.2) (Miltenyi Biotec B.V. & Co. KG, Bergisch Gladbach, Germany).

### 3.10. Statistical Analysis

A statistical analysis was performed using IBM SPSS SPSS-Inc., 2019; Reference Guide 26 SPSS, Chicago, IL, USA; and Microsoft Excel and Origin software. All data are presented as means ± SD. In brief, to evaluate the statistical significance of experimental data, comparisons between treated and control probes and cancer and normal cell lines were performed via Student’s *t*-test or the one-way ANOVA test. Each *p*-value lower than 0.05 was considered statistically significant. The obtained results were expressed in a diagram (Excel, Windows 10) and table.

## 4. Conclusions

During the last few decades, there has been an increased demand for nanodrug delivery systems capable of delivering their payloads to a specific site of action to improve the solubility and pharmacokinetic properties of the therapeutic agents, while reducing their systemic side effects. On this topic, a novel *L@F* was successfully encapsulated with natural antioxidants such us Quercetin. Our in vitro experiments performed on the breast epithelial cell lines highlighted limited cytotoxicity and the absence of antiproliferative activity in non-cancerous cells compared to cancerous ones. To the best of our knowledge, this is the first demonstration of electro-assisted delivery into breast cancer cells with alkali lignin-based formulations loaded with natural antioxidants. All these findings give us grounds to assume that the tested novel biopolymer composites, combined with electroporation, have promising potential as drug delivery systems for natural anticancer substances against drug-resistance tumors. Moreover, the proposed improved unification procedure based on size and charge, combined with the optimization of the electro-treatment protocol, open a wide field of in vitro research in view of the modification of the internalization process for overcoming limitations in the therapeutic applications of antioxidant substances of natural origin, considering their poor solubility and low bioavailability. Further studies should be performed to analyze pathways of intracellular internalization in detail and investigate hypothetical ROS-related mechanisms of apoptosis or ferroptosis in cases of combination with electroporation.

## Figures and Tables

**Table 1:** Changes in the size and ζ-Potential of all synthesized *Lignin@Formulations* before and after the separation procedure performed via Vivaspin^®^6-centrifugation. The presented data are averaged from three independent experiments for each formulation type.

Type Alkali Lignin@Formulations	Size (nm)		ζ-Potential (mV)	
	Before	After	Before	After
Lignin@F (empty)	310.70 (74.66%)	79.88 (46.78%)	−31.48	−25.35
Lignin@Naringenin F	488.70 (50.43%)	125.60 (46.39%)	−33.78	−19.37
Lignin@Morin F	361.30 (41.45%)	171.20 (42.33%)	−40.69	−32.58
Lignin@Quercetin F	413.43 (98%)	109.56 (41.97%)	−41.09	−19.89

**Table 2:** Changes in the size and ζ-potential values of synthesized *Lignin@Formulations* (empty or loaded with Quercetin) before and after applying electrical pulses with different intensities. The presented data are averaged from two independent experiments for each formulation type.

Type Electroporation Treatment	*Lignin@F (Empty)*		*Lignin@Quercetin F*	
	Size (nm)	ζ-Potential (mV)	Size (nm)	ζ-Potential (mV)
w/o Electroporation	410.6	−36.28	458.8	−33.19
After 300 V/cm	313.1	−34.56	325.4	−31.20
After 500 V/cm	379.9	−37.29	289.0	−31.66
After 700 V/cm	287.2	−37.10	252.4	−27.83
After 1000 V/cm	235.4	−26.79	269.4	−28.19

**Table 3:** Determined data of relative concentration after a unification procedure conducted via Vivaspin^®^-based centrifugation and DLS-analysis.

Formula for Calculation of Relative Concentration C^R^ = (U − Z)/(Y − Z) × X				
Parameter	Type Alkali Lignin@Formulations			
	*Lignin@* *(Empty)*	*Lignin@Naringenin*	*Lignin@Morin*	*Lignin@Quercetin*
^1^Y	−31.48	−33.78	−40.69	−41.09
^2^U	−25.35	−19.37	−32.58	−19.89
^3^X	0.05 mg/mL			
^4^Z	−10.34	−10.34	−5.06	−5.06
^5^C^R^	0.035 mg/mL	0.0247 mg/mL	0.0386 mg/mL	0.018 mg/mL

^1^Y—ξ-potential (mV) of initial supernatant phase of solution before separation procedure; ^2^U—ξ-potential (mV) of initial supernatant phase of solution after separation procedure; ^3^X—initial solution concentration in mg/mL; ^4^Z—ξ-potential (mV) of solvent, used for the initial dilution of formulations; ^5^C^R^—calculated Relative Concentration (in mg/mL) of filtrate phase solution (for the in vitro applications).

## Data Availability

Data available on request due to restrictions.

## Supplementary Materials

The following supporting information can be downloaded at https://www.mdpi.com/article/10.3390/ijms26157481/s1.
