# Supplementary material for: Alkali Lignin-Based Biopolymer Formulations for Electro-Assisted Drug Delivery of Natural Antioxidants in Breast Cancer Cells—A Preliminary Study
Source: Int J Mol Sci. 2025 Aug 2;26(15):7481. doi: 10.3390/ijms26157481 (PMC12347630; doi:10.3390/ijms26157481)

Article

Alkali Lignin-Based Biopolymer Formulations for Electro-Assisted Drug Delivery of Natural Antioxidants in Breast Cancer Cells—A Preliminary Study

Severina Semkova [1,*], Radina Deneva [1], Georgi Antov [2], Donika Ivanova [3,4,*] and Biliana Nikolova [1]

[1] Department of Electroinduced and Adhesive Properties, Institute of Biophysics and Biomedical Engineering, Bulgarian Academy of Sciences, 1113 Sofia, Bulgaria; bilianani kolova2000@yahoo.com (B.N.)
[2] Laboratory of Genome Dynamics and Stability, Institute of Plant Physiology and Genetics, Bulgarian Academy of Sciences, 1113 Sofia, Bulgaria; antov8107@abv.bg
[3] Department of Pharmacology, Animal Physiology Biochemistry and Chemistry, Faculty of Veterinary Medicine, Trakia University, 6000 Stara Zagora, Bulgaria
[4] Department of Chemistry and Biochemistry, Faculty of Medicine, Trakia University, 6000 Stara Zagora, Bulgaria
* Correspondence: severina.yordanova@gmail.com (S.S.); donika.ivanova@trakia-uni.bg (D.I.)

I. Supplementary movies (SM 1-4).

Lens-free microscopy imaging to quantify cell proliferation during 72 hours. Scale bar, 500 μm Proliferation rates, some morphological differences and cells moving fluctuations after treatments is well-shown on the recorded movies. All findings are in full correspondence to presented proliferation curves.

- **SM - 1**– untreated control cells
- **SM - 2** – Cells treated with Alkali lignin (250 μM)
- **SM - 3** – Cells treated with Quercetin (300 μM)
- **SM - 4** – Cells treated with cells treated with separated by size and charge L@Q F (calculated relative concentration: 3.5 μg/mL).

All video files could be found here:
https://drive.google.com/drive/folders/1LfDKxluDBDURiEFE4yIjnq_j9rzG-dFY?usp=drive_link

II. Supplementary Figure S1.

Flow cytometric Annexin V-FITC/PI analysis of apoptosis for the triple-negative tumorigenic highly metastatic MDA-MB-231 induced by 48 hours treatment with: Alkali lignin, Quercetin and *Lignin@Quercetin Formulations*. Cells in the lower left quadrant (Annexin V-FITC−/PI−) are viable; those in the lower right quadrant (Annexin V-FITC+/PI−) are early apoptotic and those in the upper right quadrants (Annexin V-FITC+/PI+) are late apoptotic or necrotic.

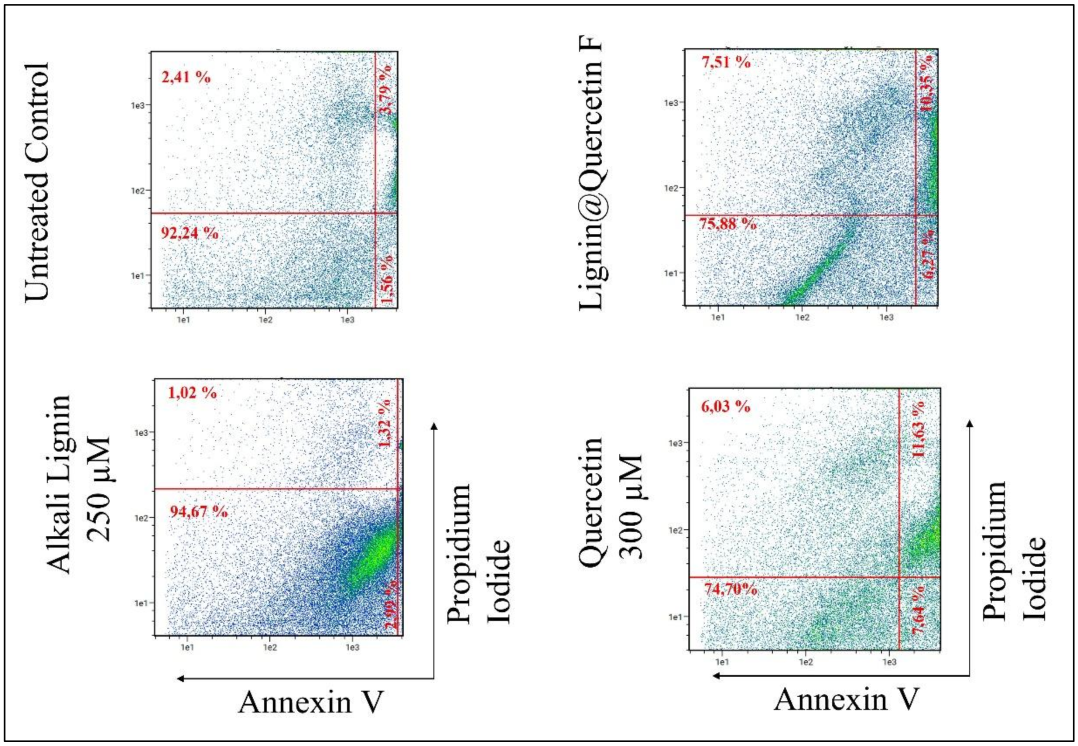

III. Supplementary Figure S2.

Cell viability of MDA-MB-231 cells incubated for 24 hours after cell culture medium volume replacement with different percent deionized Milli-Q® water. Cells grown in 100 % culture medium w/o water replacement was used as a control for calculations.

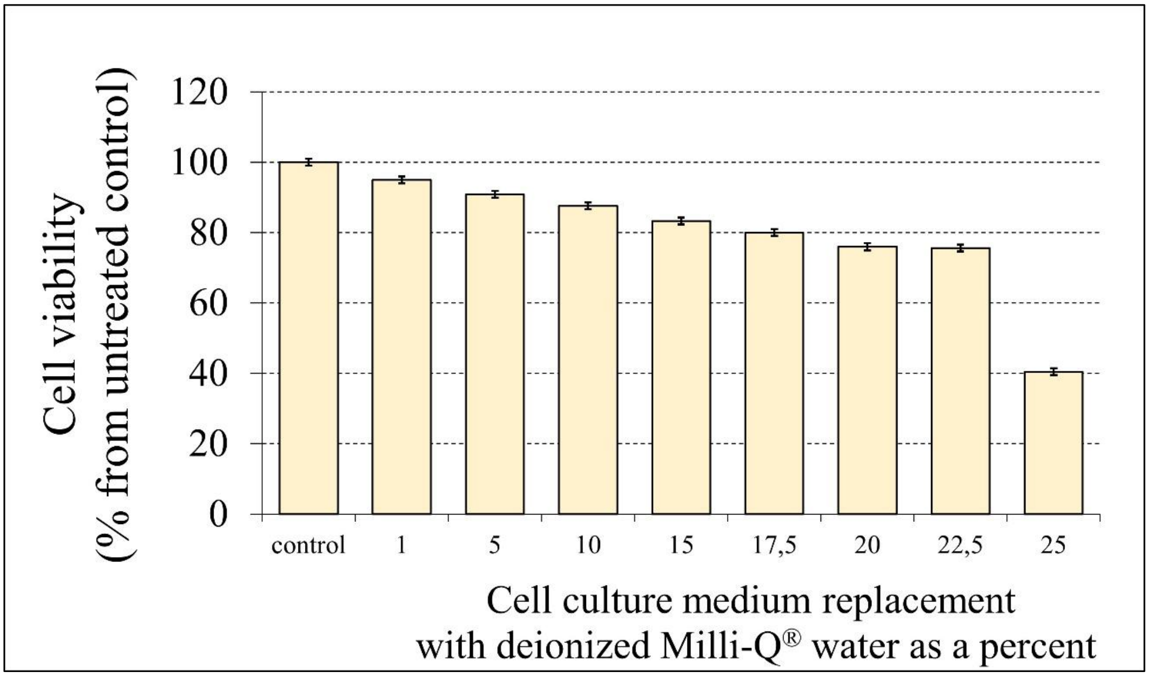

Supplement: Supplementary file 1 [file ijms-26-07481-s001.zip › ijms-3713094-supplementary.pdf]
